# How does construction of the healthcare data center affect the health of older adults?evidence from China

**DOI:** 10.3389/fmed.2025.1589319

**Published:** 2025-06-02

**Authors:** Yao Yao, Pengyu Xu

**Affiliations:** School of Economics and Management, Southeast University, Nanjing, China

**Keywords:** healthcare data center, health of older adults, digital health, health management, difference-in-differences (DID) model

## Abstract

**Background:**

The construction of the National Healthcare Data Center (NHDC) has driven data aggregation and interoperability, and created the conditions necessary for older adults to improve their health through digital health management. This study aimed to evaluate the effect of healthcare data center construction on improving the health of the older adults and elaborate on the role of health management within the underlying mechanism.

**Methods:**

The difference-in-differences (DID) model was used as an empirical strategy for causal identification. It included baseline regressions, robustness checks, heterogeneity analysis and mechanism analysis.

**Results:**

The NHDC construction significantly improves health of older adults, which is reflected in higher subjective health ratings, reduced prevalence of chronic diseases and multimorbidity, enhanced physical function, and improved mental health. Heterogeneity analysis indicates that the effect is more significant among rural residents, individuals under 60 years of age, and male seniors. Increased active health management efforts by the older adults, along with a higher likelihood of receiving health management services, play a significant role in the channels of effect.

**Conclusion:**

The construction of the healthcare data center characterized by driving data aggregation and interoperability improves the health of older adults by promoting their digital health management.

## Background

With declining fertility rates and increasing life expectancy, China is facing significant challenges posed by population aging. Research shows that older adults in China generally experiences longevity accompanied by poor health, with over 78% of older adults suffering from at least one chronic disease (1). As a result, the health of older adults has become a critical social issue in China.

Over the past two decades, there has been an exponential growth in healthcare data worldwide. The rapid iteration of digital technologies has accelerated the progress of health informatization, digitization, and smart healthcare, the rapid development of online health communities (2) and the widespread use of wearable devices (3), which has enabled hospitals, community health service centers, health examination institutions, and medical IT enterprises in China to accumulate vast amounts of healthcare data, creating an unprecedented volume of analyzable information (4). Against this backdrop, digital health based on healthcare data, characterized by mobile health, telemedicine, and personalized medicine, has begun to play a transformative role, finding broad applications in disease diagnosis and treatment, chronic disease management, and epidemic prevention and control. Digital health has thus emerged as an innovative solution to address the challenges of the health of older adults.

To promote the development and application of healthcare data and adapt to new trends in digital health, China’s National Health Commission initiated the Pilot Project of the National Healthcare Data Center (NHDC) in 2016, and three batches of pilot provinces have carried out this work between 2016 and 2023. Shortly after the implementation of the pilot program, pilot provinces accelerated the planning and policy support for data center construction (5). Specifically, they have implemented a series of policy practices around four key themes: data aggregation, data interoperability, “data sinking,” and data-driven empowerment. These practices include enhancing healthcare data repositories, promoting data and information sharing across healthcare institutions, addressing gaps in health informatization and digitization of grassroots, and improving digital health service capabilities. While older adults gain access to digital health services, they also benefit from the health improvement effects brought about by the construction of the NHDC. But what exactly are these effects on the health of older adults? What are the underlying theoretical mechanisms? These questions warrant in-depth evaluation and study.

Previous studies have demonstrated that access to and utilization of traditional internet services positively impact health outcomes through channels such as improving the accessibility of health information and enhancing quality of life (6). These studies primarily emphasize the effects on individuals, or more specifically, the demand side of health services. However, under the new wave of digitalization, an increasing body of research indicates that digital health can also influence the supply side of health services, enabling the provision of higher-quality services and thereby improving individual health outcomes. For instance, the development of digital infrastructure enhances the accessibility of public health services (7); artificial intelligence can rapidly and accurately analyze large-scale medical data, such as medical imaging and pathological slides, assisting physicians in disease diagnosis and significantly improving treatment outcomes (8). Additionally, the widespread adoption of electronic health record (EHR) systems allows physicians to access and track patients’ complete medical histories, including diagnoses, treatments, and medication usage, thereby reducing the risk of undetected disease progression (9).

Despite these advancements, there remains a gap in the academic literature concerning the role of healthcare data aggregation and interoperability in influencing both the supply and demand sides of health services, and how these interactions collectively impact health outcomes. This study sought to address this gap by examining the effects of healthcare data aggregation and interoperability on the health of older adults within the context of digital health.

Moreover, research suggests that the greatest role of digital health lies not in treatment but in prevention (10). Intervention models and strategies supported by healthcare data have become a new trend in health management (11). Data-driven smart health management is more convenient and personalized, encouraging more people to improve their physical condition through health management and accelerating the interoperability of digital health and preventive medicine into daily life. Health applications and online health platforms, combined with smart wearable devices, not only provide individuals engaging in health management with abundant health knowledge and resources but also foster good health habits. This comprehensive improvement in health literacy contributes to the effective prevention of chronic diseases among the older adults (12). Therefore, when examined from the perspective of disease prevention, the health improvement effects of healthcare data center construction on older adults are significant, which calls for empirical validation.

Based on this context, this study tried to analyze the effect of healthcare data center construction, particularly in promoting data aggregation and interoperability, on improving the health of the older adults. Theoretical framework was established to clarify the underlying mechanisms and analyze the role of health management from a perspective of prevention. And a difference-in-differences (DID) approach was employed for empirical testing. The remainder of this study was structured as follows: the second section explained the theoretical mechanisms and proposed research hypotheses. The third section introduced the empirical research design, including identification strategy, data, and variables. The fourth section presented the empirical analysis results. The fifth section provided further discussion on the findings, policy implications, and limitations. The sixth section concluded this study.

### Theoretical mechanism and research hypothesis

Based on existing theoretical research and policy practices regarding the NHDC, we systematically analyzed the mechanisms through which the pilot project of the NHDC influence the health of older adults, and proposed research hypotheses accordingly.

Firstly, pilot provinces have issued various plans and supportive policies to strengthen the construction of the NHDC, thereby encouraging deliberate collection and application of healthcare data across the region. This creates an innovation-friendly environment in the medical and health sector that leverages data as a fundamental factor, facilitating the widespread diffusion and adoption of novel digital health technologies, models, and business formats in pilot regions. Drawing from the theories of innovation diffusion and social network contagion (13, 14), healthcare institutions, enterprises in the health industry, digitally skilled healthcare professionals, and certain early-adopting patients serve as the diffusion sources of digital health. Online healthcare platforms and health-related applications function as effective diffusion channels that accelerate penetration. Older adults can leverage health information and digital health technologies to enhance health behaviors, perform self-diagnoses, and make informed treatment decisions, thereby improving their overall health status (15).

Secondly, the construction of the NHDC facilitates the collection, storage, and interoperability of healthcare data, addressing the issues of “data silos” and “data stovepipes” caused by fragmented and unconnected datasets. This drives a more balanced allocation of physical and virtual medical resources, which were previously unevenly distributed (16). Pilot regions’ healthcare institutions can utilize health information platforms and centralized data repositories to achieve interconnectivity and data sharing within the region, thereby fostering information coordination and enhancing the flow efficiency of virtual medical resources. Data interoperability between higher-level medical institutions and grassroots healthcare facilities within medical alliances strengthens internal cooperation, enabling integrated tiered healthcare services that link primary care with higher-level facilities. This addresses the shortcomings in grassroots healthcare data infrastructure and medical resource availability, ultimately improving the capacity of primary healthcare services (17). Rational resource allocation alleviates challenges in healthcare access and the limited supply of grassroots healthcare services, encouraging older adults to make better use of primary healthcare services (18), thus exerting a positive impact on their health.

Thirdly, the healthcare data center integrates health knowledge, optimize service efficiency, and enhances digital health service capabilities, thereby increasing both the quantity and quality of health services (19). Moreover, given the positive externality of data as a production factor, the aggregation of healthcare data often generates spillover effects, resulting in additional health services. For instance, during the establishment of residents’ EHRs, a range of complimentary health services may be provided. Theoretically, the stronger the accessibility of medical resources, the easier it is for individuals to obtain healthcare services, leading to more pronounced health benefits (20). In this context, the older adults gain access to an increased quantity and improved quality of health services, which may encourage greater utilization and subsequently improve their health status (21). Based on the above, Hypothesis 1 is proposed:

1. The construction of the healthcare data center improves the health outcomes of older adults.

Next, from a research perspective of prevention, we explored the role of health management in the mechanisms.

On the one hand, personal healthcare data constitute a significant component of the healthcare data stored in the NHDC. Pilot policies explicitly emphasize accelerating the collection of personal healthcare data, thereby fostering the adoption of personal smart wearable devices and dynamic health monitoring technologies. The diffusion of such devices among elderly populations not only provides physical support for their health management (3), but also incorporates innovative intervention models. These models include data monitoring requirements integrated into physicians’ medical advice, continuously updated health information through remote doctor-patient interactions, and incentive mechanisms by insurance companies based on wearable devices. These factors may guide older adults to proactively improve health behaviors such as maintaining a healthy diet, engaging in sufficient exercise, quitting smoking, and moderating alcohol consumption. Additionally, they foster awareness of health monitoring and encourage regular health checks (22). Improvements in such health behaviors can be regarded as an increased effort in active health management, which may enhance health outcomes for the older adults (23). Based on the above, Hypothesis 2 is proposed:

2. The construction of the healthcare data center promotes active health management among the older adults, thereby improving their health outcomes.

On the other hand, the construction of regional health information platforms, a key component of the NHDC pilot program (24), is closely linked to chronic disease management systems. Coupled with strengthened data collection, circulation, and analysis enabled by the healthcare data center, information coordination between healthcare institutions begins to manifest its impact on health management. First, new models such as online appointment scheduling, intelligent referral systems, and remote health management improve the efficiency with which older adults receive health management services. Second, the sharing and interoperability of healthcare data, along with intelligent performance evaluations, enhance the efficiency of health management services delivered by grassroots healthcare systems and family doctor teams. These improvements influence health management services from both supply and demand perspectives, ultimately exerting a positive impact on the health of older adults.

The regional health information platform has also accelerated the development and application of contract-based service information systems. Health information providers utilize online platforms, such as mobile apps, to offer contracted older adults services like online enrollment, health consultations, appointment scheduling, health management, chronic disease follow-ups, and access to medical reports. This online service model further empowers free health examinations and chronic disease management services by family doctors. Moreover, the development of comprehensive population information databases and EHRs necessitates that grassroots healthcare institutions establish health records for all residents within their jurisdictions. These EHRs mandate monitoring of key populations, resulting in spillover benefits such as health checks, chronic disease prevention, and monitoring services. In this context, the availability and quality of health management services surrounding older adults are significantly enhanced. Consequently, the older adults are likely to increase their utilization of health management services, thereby improving their health status. Based on the above, Hypothesis 3 is proposed:

3. The construction of the healthcare data center increases the probability of older adults accessing external health management services, thereby improving their health outcomes.

## Materials and methods

### Data source

The data used in this research was sourced from the China Health and Retirement Longitudinal Study (CHARLS). To meet the research needs, we combined panel data from four survey waves: 2013, 2015, 2018, and 2020. Demographic, economic and health-related data at the provincial level were obtained from the provincial Statistical Yearbooks and Health Statistical Yearbooks of China. We merged the provincial data and individual data in CHARLS to get the final sample. Although in China, the older adults are defined as 60 years of age and above in relevant Chinese laws, to include the population born during the “baby boom” period (1962–1969), which is approaching old age, the sample was selected from the population aged over 50, including some middle-aged people. After excluding observations with missing or abnormal values in key variables, as well as samples of individuals aged 50 years and below, we obtained 37,034 valid observations.

### Variable description

The primary independent variable in this study was healthcare data center construction, measured by the dummy variable of the NHDC, it takes the value of 1 when a respondent’s current city of residence was impacted by the pilot project and 0 otherwise.

The dependent variable in this study is the health of older adults, measured using the indicator of the self-rated health, which relates to the question in the CHARLS questionnaire: “How do you feel about your health?” Answers were given on a scale of 1–5, with 1 being the worst health and 5 being the best. We used the indicator in baseline regression, robustness tests and mechanism tests. To minimize the potential bias and one-sidedness of the subjective indicator, as it may be influenced by respondents’ emotions (25), we also evaluated health of older adults through multiple dimensions such as chronic diseases, physical function, and mental health.

For chronic diseases, the CHARLS questionnaire covers 14 chronic conditions. If respondents report being diagnosed with any of these, they are considered chronic disease patients. Given the consensus in recent literature that chronic diseases rarely exist in isolation (26, 27), we simultaneously considered comorbid chronic diseases as indicators of the health of older adults.

Physical function was assessed using Activities of Daily Living (ADL), which includes six activities covered in the CHARLS questionnaire: dressing, eating, bowel and bladder control, toileting, bathing, and bed-chair transfer. Based on the respondent’s score, ADL capabilities are classified into: independent (100), mildly dependent (75–95), moderately dependent (50–70), severely dependent (25–45), and completely dependent (0–20).

Mental health was measured using the Center for Epidemiologic Studies Depression Scale (CES-D), where the CHARLS questionnaire includes 10 items from the scale. The lower the score, the lower the level of depression, indicating better mental health.

Control variables were selected at both regional and individual levels. At the regional level, we considered variables such as per capita GDP, the number of practicing physicians per 10,000 people, and the number of hospital beds per 10,000 people to reflect regional demographic, economic and medical conditions. At the individual level, control variables include age, sex, marital status, education level, and place of residence.

Mediating variables were measured primarily in terms of both individual active health management and external health management services for older adults. Individual active health management includes both health behavior improvement and active health monitoring. Health behaviors were represented by dummy variables based on questions from the CHARLS questionnaire related to drinking, smoking, and exercise, indicating whether the respondent drinks, smokes, or exercises. Active health monitoring was represented by a dummy variable based on questions on blood pressure or blood glucose checks, indicating whether the respondent actively measure blood pressure and blood glucose and other indicators. For external health management services, we identified whether the older adult received physical examinations services based on questions “Have you exercised in the past year,” and identified whether the older adult received chronic disease management services based on questions “Has a doctor ever taken your blood pressure/blood glucose?” and “Has a doctor advised you to control weight, exercise, adjust diet, quit smoking, etc.,?” (1). EHR related health services were difficult to measured, since grass-roots organizations in various regions adopt different methods when establishing EHR within their jurisdictions, making it difficult to conduct standardized measurement of the services. We noticed that chronic disease patients are prioritized for EHR, with specific guidelines and records in the *Resident Health Management Service Specifications* in China. Therefore, we focused on chronic disease patients here: if they have ever had a blood pressure check by a doctor, received relevant services from primary healthcare institutions, or received home visits by a doctor, they were considered to have an EHR and receive health services in the process of creating the EHR.

Detailed variable definitions and descriptive statistics are shown in Table 1.

**TABLE 1:** Variable definitions and descriptive statistics.

Variables	Definitions	Mean/percentage	S.D.	Max	Min
**Explanatory variable**					
NHDC	1 if the respondent’s current city of residence was impacted by the pilot project of the NHDC, otherwise 0.	0.07	0.26	0	1
**Dependent variables**					
Health	1–5, 1 indicates that the respondent’s self-rated health is the worst, and five indicates the best.	2.74	1.08	1	5
Chronic	1 if the respondent has a chronic disease, otherwise 0.	0.73	0.44	0	1
Chronic_co	1 if the respondent has a chronic disease co-morbidity, otherwise 0.	0.52	0.50	0	1
ADL	0–100, 20 and below indicates that the respondent is completely dependent on others in daily lives, and 100 indicates complete independence.	93.17	11.94	0	100
CES_D	0–3, 3 indicates that the respondent has no depressive symptoms, and 0 indicates significant depressive symptoms.	1.31	1.12	0	3
**Control variables**					
Age	Age of the respondent.	62.76	8.13	51	97
Sex	1 if the respondent is male, otherwise 0.	0.50	0.50	0	1
Marital	1 if the respondent has a spouse, otherwise 0	0.86	0.35	0	1
Edu	1–4, 1 indicates that the respondent has an elementary school education or less, 2 indicates middle school, 3 indicates high school, and 4 indicates college education or above.	1.51	0.78	1	4
Urban	1 if the respondent lives in an urban area, otherwise 0.	0.25	0.44	0	1
Lnpgdp	Log of GDP per capita in the region where the respondent lives.	10.79	0.35	10.00	12.01
Doc	Number of physicians per 10,000 people where the respondent lives.	23.50	5.10	13	59
Bed	Number of beds in medical institutions per 10,000 people where the respondent lives.	54.47	10.05	35.55	79.50
**Mediating variables**					
Drinking	1 if the respondent has drunk alcohol in the past year, otherwise 0.	0.36	0.48	0	1
Smoking	1 if the respondent has smoked in the past year, otherwise 0.	0.32	0.47	0	1
Exercise	1 if the respondent has exercised in the past year, otherwise 0.	0.40	0.49	0	1
Monitoring	1 if the respondent has actively measured blood pressure and blood glucose and other indicators in the past year, otherwise 0.	0.91	0.29	0	1
PE	1 if the respondent has received physical examination services in the past year, 0 otherwise.	0.42	0.49	0	1
HM	1 if the respondent has received chronic disease management services in the past year, 0 otherwise.	0.60	0.49	0	1
EHR	1 if the respondent has received health services related to EHR establishment in the past year, 0 otherwise.	0.90	0.31	0	1

### Empirical strategy

To clarify how the construction of the healthcare data center affects the health of older adults, we considered the NHDC pilot project as a policy shock to construct a quasi-natural experiment, then the following time-varying DID model was employed:


(1) $$Y_{ijt}=\beta_{0}+\beta_{1}{\text{NHDC}}_{ijt}+\beta_{2}{\text{Control}}_{ijt}+\mu_{j}+\gamma_{t}+\varepsilon_{ijt}$$

where subscripts i, j, and t represent individuals, regions and years, respectively. *Y*_*ijt*_ denotes the health of older adults, measured using a series of health-related indicators. *NHDC*_*ijt*_ is a policy dummy variable representing the construction of healthcare data center and β_1_ is the main coefficient of interest. *Control*_*ijt*_ refers to the individual- and region-level control variables. μ_*j*_ and γ_*t*_ are the fixed effects for region *j* and year *t*, and ε_*ijt*_ is the random error term. We used the model for the baseline regression and examined the validation of the DID model through several robustness tests.

To empirically examine the potential mechanism by which health management mediates the impact of the healthcare data center construction on the health of older adults, we employed the following models:


(2) $$\begin{array}{c} M_{ijt}=\beta_{0}+{\beta^{\prime }}_{1}NHDC_{ijt}+\beta_{2}{\text{Control}}_{ijt}+\mu_{j}+\\ \gamma_{t}+\varepsilon_{ijt} \end{array}$$

(3) $$\begin{array}{c} Y_{ijt}=\beta_{0}+{\beta^{\prime \prime }}_{1}NHDC_{ijt}\times M_{ijt}+\beta_{2}{\text{Control}}_{ijt}+\\ \mu_{j}+\gamma_{t}+\varepsilon_{ijt} \end{array}$$

In equation (2) and (3), *M*_*ijt*_ represents the mediating variable of health management; β′_1_ indicates the degree to which the NHDC influences health management of older adults, while β″_1_ represents the impact of the NHDC construction on health of older adults through its effect on health management. The remaining variables have the same meanings as in the equation (1). Equation (2) investigates whether the construction of the NHDC affects health management of older adults, while Equation (3) incorporates an interaction term between the policy dummy variable (*NHDC*_*ijt*_) and the mediating variable (*M*_*ijt*_) to further examine its impact on health of older adults.

## Results

### Baseline regression

We first employed model (1) for the baseline regression and used a DID method to estimate the impact of healthcare data center construction on the health of the older adults. The results are shown in Table 2: From columns (1) to (5), the findings indicate that the implementation of the NHDC significantly improved the health status of the older adults in pilot province. Specifically, column (1) shows that self-rated health of older adults affected by the pilot project increased by 0.128 units compared to the control group, which is statistically significant at the 1% level. Columns (2) and (3) demonstrate that the NHDC significantly reduced the probability of older adults suffering from chronic diseases and chronic disease co-morbidities in pilot provinces, by 4.2% and 2.6%, respectively. Columns (4) and (5) showed that the NHDC contributed to the improving of older adults’ activities of daily living and the reducing of their tendency toward depression.

**TABLE 2:** Baseline regression results.

Variables	(1)	(2)	(3)	(4)	(5)
	Health	Chronic	Chronic_co	ADL	CES_D
NHDC	0.128***	−0.042***	−0.026**	0.735**	0.089*
	(5.361)	(−4.431)	(−2.433)	(2.505)	(1.810)
Age	−0.010***	0.007***	0.009***	−0.293***	0.003***
	(−9.152)	(11.212)	(14.128)	(−24.011)	(2.833)
Sex	0.143***	−0.060***	−0.070***	3.115***	0.352***
	(11.002)	(−5.437)	(−6.113)	(13.378)	(25.581)
Marital	−0.005	0.016	0.011	0.910***	0.127***
	(−0.235)	(1.605)	(1.234)	(4.573)	(6.435)
Edu	0.077***	−0.009*	−0.007	0.996***	0.031**
	(14.648)	(−1.866)	(−1.257)	(8.470)	(2.718)
Urban	0.142***	0.029***	0.045***	1.721***	0.132***
	(5.183)	(2.993)	(4.087)	(9.334)	(6.559)
Lnpgdp	0.122	−0.072	0.096*	−2.568*	0.288
	(0.827)	(−1.456)	(1.997)	(−1.727)	(1.327)
Doc	0.004	−0.000	0.002	0.067	−0.012*
	(0.929)	(−0.286)	(1.299)	(1.294)	(−1.919)
Bed	−0.011**	0.002*	0.001	0.016	0.001
	(−2.693)	(1.732)	(0.931)	(0.446)	(0.128)
Constant term	2.366	0.983*	−1.135**	132.471***	−2.105
	(1.571)	(1.843)	(−2.254)	(8.634)	(−0.949)
Fixed effects	Yes	Yes	Yes	Yes	Yes
Observations	37034	37034	37034	37034	37034
Adj R2	0.103	0.049	0.063	0.101	0.070

*,

**,

*** show significant at the 10%, 5%, and 1% levels, respectively; t-values in parentheses.

### Robustness tests

**FIGURE 1:**
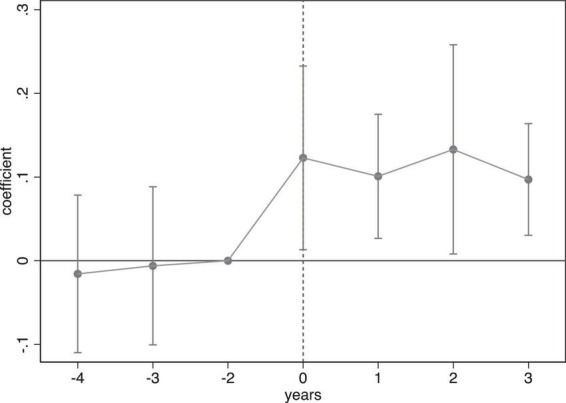
Parallel trend test.

To validate the use of the DID method, it’s essential that the treatment and control groups exhibit similar trends before the policy was implemented. We used an event study approach, setting the two periods prior to the NHDC as the baseline. The results, depicted in Figure 1, show no significant trend differences in self-rated health between the treatment and control groups prior to the pilot project, confirming that the baseline regression passed the parallel trend test. In addition, we could learn from Figure 1 that the health improvement effects of the NHDC rose rapidly immediately after the pilot program was initiated, peaked 2 years later, and then began to attenuate—though they remained better than pre-pilot levels. A possible explanation is that as digital health accessibility continued to increase, the primary limiting factor for improving health of older adults through digital technology shifted to older adults’ digital literacy, leading to diminishing marginal effects of further enhancements to digital health infrastructure.

**FIGURE 2:**
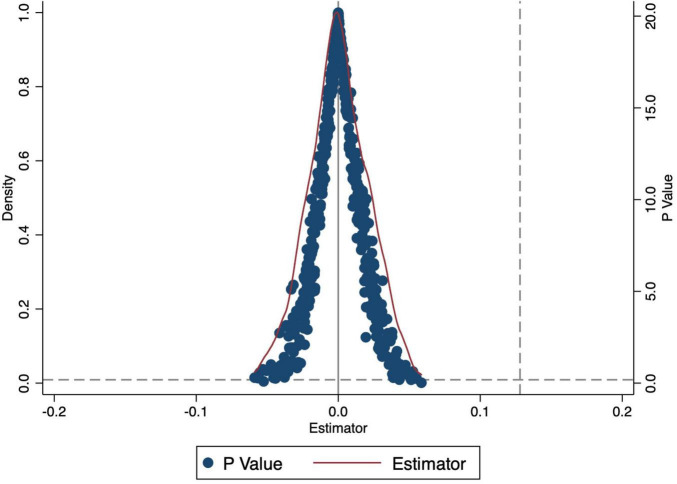
Placebo test.

To verify whether the baseline results were influenced by unobserved factors, we conducted a placebo test through randomly selecting pilot provinces and implementation times to construct new policy dummy variables and repeating the regression process 500 times. The distribution of estimated coefficients, shown in Figure 2, mostly clustered around zero, with few reaching the significance level of the real coefficient (0.128). This further substantiates the robustness of the results from the baseline regression.

**FIGURE 3:**
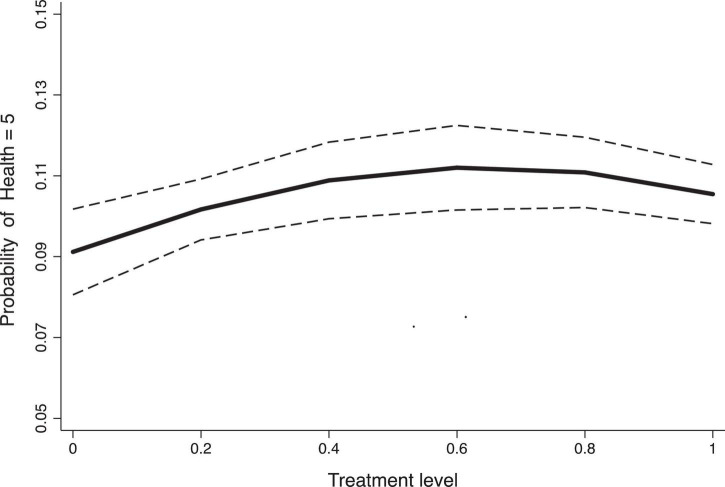
Dose response function.

Additionally, we attempted to measure the dynamic effects of the NHDC pilot using a dose-response function. Since the variable NHDC is a non-continuous categorical variable, we could not directly observe its dose-response effects. To address this, we used the completion rate of county-level health information platforms in pilot regions as a proxy for NHDC intensity. Building such platforms was an explicit task of the NHDC pilot, and we obtained data on platform completion rates across regions from 2017 to 2020, allowing us to construct a continuous variable reflecting pilot intensity. We adopted the Generalized Propensity Score (GPS) method and re-estimated treatment effects under varying policy intensities using an Ordered Logit Model, with 90% confidence intervals calculated via the bootstrap method. The dose-response function is plotted in Figure 3, where the solid line represents the treatment effect, and the two dashed lines denote the upper and lower bounds. The trend of the function aligns closely with the temporal policy effects observed in the event study analysis, indicating an attenuation effect in treatment intensity as the pilot progresses.

The selection of pilot provinces may not have been random, and the older adults in pilot and non-pilot regions might have inherent differences. To account for these factors, propensity score matching difference in difference (PSM-DID) analysis was used to control for confounding factors. Specifically, we estimated propensity scores using covariates in a logit model, employed a 1:1 nearest-neighbor matching method with a caliper of 0.05, and re-estimated the DID model using the matched sample. As shown in column (1) of Table 3, the coefficient is 0.108 and is significant at the 5% level, supporting the robustness of the findings.

**TABLE 3:** Robustness tests.

Variables	(1)	(2)	(3)	(4)	(5)
	Health	Health	Health	Health	Health
NHDC	0.108**	0.141***	0.135***	0.140***	0.127***
	(2.372)	(6.099)	(5.370)	(3.642)	(4.140)
Treat_1	–	0.034	–	–	–
	–	(1.070)	–	–	–
Treat_2	–	–	0.059**	–	–
	–	–	(1.801)	–	–
Control	Yes	Yes	Yes	Yes	Yes
Fixed effects	Yes	Yes	Yes	Yes	Yes
Observations	22,332	37,034	37,034	12,716	37,034
Adj R^2^	0.098	0.103	0.103	0.030	0.116

*,

**,

*** show significant at the 10%, 5%, and 1% levels, respectively; t-values in parentheses.

To prevent other concurrent policies related to big data or health of older adults from affecting the results, we added dummy variables for relevant policies, such as the National Big Data Comprehensive Experimental Zones (*Treat_1*) and National Integrated Medical Care Service Pilot (*Treat_2*), following previous studies (1, 28). As seen in columns (2) and (3) of Table 3, after controlling for the effects of these concurrent policies, the results remained robust.

The baseline regression used a sample of older adults aged 50 and above. To test for any potential age bias, we reran the regression using a sample of older adults aged 65 and above. The result in column (4) was still significantly positive, indicating that the sample selection based on the respondent’s age does not introduce any specific bias.

To eliminate any potential interference from city-level heterogeneity, we replaced the province fixed effects in the baseline model with city fixed effects. According to the result in column (5), the main results in baseline regression remained unaffected.

### Heterogeneity analysis

The empirical research continued by four subgroup regressions to examine whether the construction of the healthcare data center impacted health of older adults differently based on regional and individual characteristics. To address potential bias in directly comparing coefficients across different groups in the regressions, we applied Fisher’s Permutation Test to assess coefficient differences between groups (29). If the test results are significant, it indicates that the coefficients are comparable across groups. The results of subgroup regressions are summarized in Table 4.

**TABLE 4:** Heterogeneity analysis.

Variables	Panel A		Panel B	
	(1)	(2)	(3)	(4)
	**Urban sample**	**Rural sample**	**Younger sample**	**Elder sample**
NHDC	−0.020	0.167***	0.193***	0.114***
	(−0.810)	(5.839)	(3.761)	(3.493)
Control	Yes	Yes	Yes	Yes
Fixed effects	Yes	Yes	Yes	Yes
Observations	9,390	27,644	18,142	20,928
Adj R^2^	0.082	0.111	0.129	0.084
*P*-value of Fisher’s permutation test	0.000***		0.098*	
**Variables**	**Panel C**		**Panel D**	
	**(5)**	**(6)**	**(7)**	**(8)**
	**Male sample**	**Female sample**	**Internet access sample**	**No internet sample**
NHDC	0.193***	0.076**	0.211**	0.005
	(5.331)	(2.544)	(2.612)	(0.039)
Control	Yes	Yes	Yes	Yes
Fixed effects	Yes	Yes	Yes	Yes
Observations	18,527	18,507	24,071	12,963
Adj R^2^	0.105	0.098	0.117	0.107
*P*-value of Fisher’s permutation test	0.010**		0.000***	

*,

**,

*** show significant at the 10%, 5%, and 1% levels, respectively; t-values in parentheses.

Firstly, the results of regressions based on the older adults’ residence location in panel A show that the NHDC significantly improved health only for rural elderly populations. This effect may be because the construction of NHDC enhanced healthcare data interoperability among medical institutions, addressing rural areas’ limitations in health information technology. This potentially narrows the healthcare resource gap between rural and urban areas, allowing rural older adults to access better healthcare services, while this effect has not yet appeared in urban areas.

Secondly, the results in panel B and panel C also reveals that the NHDC has a more substantial positive effect on the health of older adults aged under 60 and on male seniors. Specifically, the coefficient for both groups is 0.193, significant at the 1% level, which is higher than the 0.114 for the over 60 age group and 0.076 for females. This may be because individuals aged 50–60 have more opportunities to interact with digital technologies through social interactions and work, whereas those over 60 may experience reduced social engagement post-retirement, potentially leading to a more closed mindset. Similarly, compared to female, male seniors are more willing to seek external support to maintain their health (30) and tend to adopt digital health technologies faster. In contrast, female seniors may face more significant challenges with digital literacy (31). As a result, male seniors and seniors under 60 may benefit more from the health advantages brought by NHDC.

Thirdly, the sample in panel D was divided based on whether the older adults had broadband internet access at their residence. The results show that the NHDC significantly improved health only for those older adults with internet access, with a coefficient of 0.211. This further suggests that the positive health impact of the NHDC requires the older adult to have digital access and a certain level of digital literacy.

### Mechanism analysis

Based on theoretical analysis presented earlier, the construction of the NHDC might encourage more older adults in pilot areas to engage in proactive health management and increase their likelihood of accessing external health management services, thereby impacting their health status. We sought to empirically validate this mechanism based on Models (2) and (3).

The construction of the NHDC might enhance the willingness of older adults to engage in proactive health management, primarily by encouraging improved health behaviors and fostering awareness among the older adults to actively monitor health indicators. The results of the mediating mechanism analysis regarding individual active health management were shown in Table 5: the results in columns (1)–(3) indicate that the NHDC’s effect on improving health behaviors of older adults is mainly reflected in increased exercise participation, while it does not significantly impact smoking and drinking habits. The results in column (3a) show that exercise behavior promoted by the NHDC implementation has led to an improvement in self-rated health by 0.055 units for this subset of the older adults. This suggests a potential for older adults to use digital health technologies to manage their health and thereby enhance their health levels. Additionally, the results in columns (4) and (4a) confirm that the NHDC construction facilitates proactive monitoring of health indicators among older adults, resulting in a 0.072-unit increase in self-rated health.

**TABLE 5:** Mechanism analysis (individual active health management as the mediator).

Variables	Health behaviors improvement				Active health monitoring	
	**(1)**	**(2)**	**(3)**	**(3a)**	**(4)**	**(4a)**
	**Drinking**	**Smoking**	**Exercise**	**Health**	**Monitoring**	**Health**
NHDC	0.014	0.005	0.097**	–	0.022**	–
	(1.095)	(0.450)	(2.546)	–	(2.532)	–
NHDC*Exercise	–	–	–	0.055**	–	–
	–	–	–	(2.156)	–	–
NHDC*Monitoring	–	–	–	–	–	0.072***
	–	–	–	–	–	(2.909)
Control	Yes	Yes	Yes	Yes	Yes	Yes
Fixed effects	Yes	Yes	Yes	Yes	Yes	Yes
Observations	37004	34064	23727	23727	9596	9596
Adj R^2^	0.207	0.382	0.117	0.112	0.081	0.067

*,

**,

*** show significant at the 10%, 5%, and 1% levels, respectively; t-values in parentheses.

Moreover, the construction of the NHDC might enable older adults in pilot areas to receive more health management services from external sources such as healthcare institutions and family doctors. These health management services primarily included physical examination, chronic disease management, and EHR-related services. The results of the mediating mechanism analysis for external health management services were shown in Table 6: the construction of the NHDC in pilot areas has increased the likelihood of older adults receiving health management services, including physical examination services, chronic disease management services, and EHR-related services, all of which significantly improve the health of adults.

**TABLE 6:** Mechanism analysis (external health management services as the mediator).

Variables	Physical Examination Service		Chronic Disease Management Services		EHR-Related Health Services	
	**(5)**	**(5a)**	**(6)**	**(6a)**	**(7)**	**(7a)**
	**PE**	**Health**	**HM**	**Health**	**EHR**	**Health**
NHDC	0.049*	–	0.053*	–	0.086***	–
	(1.734)	–	(2.011)	–	(7.578)	–
NHDC*PE	–	0.123**	–	–	–	–
	–	(2.671)	–	–	–	–
NHDC*HM	–	–	–	0.070**	–	–
	–	–	–	(2.144)	–	–
NHDC*EHR	–	–	–	–	–	0.135***
	–	–	–	–	–	(6.617)
Control	Yes	Yes	Yes	Yes	Yes	Yes
Fixed effects	Yes	Yes	Yes	Yes	Yes	Yes
Observations	36,250	36,250	11,349	11,349	13,027	13,027
Adj R^2^	0.089	0.107	0.030	0.066	0.030	0.061

*,

**,

*** show significant at the 10%, 5%, and 1% levels, respectively; t-values in parentheses.

## Discussion

The NHDC pilot project is a key initiative for China to promote the interoperability and implementation of the two strategies of Healthy China and Digital China. The construction of the NHDC has created the conditions necessary for older adults to improve their health through digital health management.

This study finds that the NHDC construction significantly improves health outcomes of older adults, and increased active health management efforts by the older adults, along with a higher likelihood of receiving health management services, play a significant role in the channels of effect. The results reveal that the construction of the healthcare data center characterized by driving data aggregation and interoperability can improve the health of older adults by promoting their digital health management.

The findings validate the efficacy of a data-driven digital healthcare system from a preventive perspective. This system enhances the utilization of data resources through digitized medical services (e.g., disease surveillance), providing comprehensive health management for older adults. The system’s effectiveness extends beyond China - international evidence demonstrates that data-driven healthcare systems address seniors’ multidimensional needs (medical care, psychological support, home safety) across developed nations, each developing distinctive smart healthcare models.

In 2009, the United States government empowered the ONC (Office of the National Coordinator for Health IT) to facilitate health data sharing, accelerating digital healthcare adoption. American tech giants have advanced wearable technologies – Intel’s “A-Wear” project enables real-time health monitoring through big data analytics (32). The United Kingdom NHS integrates smart home devices to ensure elderly safety via gas leak detection, intrusion alerts, medication adherence monitoring, and fall detection. NHS’s centralized health information platform, aggregated on data.gov.uk, exemplifies large-scale medical data integration (33). Japan prioritizes assistive robotics (e.g., Paro therapeutic robots for companionship and care (34) and advanced medical imaging infrastructure. Its National University Hospital Medical Data Backup System employs SINET and L2VPN technologies to consolidate clinical data from 46 institutions, building foundations for future big-data analytics.

International studies confirmed these systems’ health impacts. For comorbidity management, remote monitoring platforms like Omada Health reduce hospitalization risk by 18% in diabetic patients with cardiovascular complications through multimodal data tracking (blood glucose, blood pressure). The NHS “Digital First” program, integrating wearables with EHRs, decreased emergency visits by 23% among COPD patients with depression (35). These cross-national experiences demonstrate the transformative potential of digital healthcare ecosystems in aging societies.

### Contribution

The contributions of this study are as follows: First, this study conceptualized the construction of the healthcare data center as a large-scale digital health access initiative, grounded in data aggregation and interoperability, with the goal of improving population health. It demonstrated that the impact of such initiatives on individual health is both direct and multifaceted. By systematically elucidating the theoretical mechanisms through which healthcare data center construction influences the health of older adults and conducting empirical testing, this study enriched the theoretical and empirical literature on the effects of digitalization on individual health. Second, research on the effects of the NHDC remains scarce. The only existing study examined the impact of the pilot project on innovation in the pharmaceutical and healthcare industries. This study provided empirical evidence on the health improvement effects of the pilot project, contributing to the understanding of the social benefits of healthcare data center construction and offering a theoretical foundation for policy refinement. Finally, this study focused on health management driven by the construction of the healthcare data center within the context of emerging digital health trends, and not only examined health management services received by the older adults but also incorporated the active health management efforts of this demographic. This dual perspective advanced the empirical research on health management and provided innovative insights into this field.

### Implications for policy and practice

This study has several implications. First, from the inception of the NHDC pilot project, the goal was to enhance public health and welfare. The main results of this study confirm the health benefits brought by the pilot policy, demonstrating the effectiveness of regional healthcare data center construction and the substantial potential value of healthcare data. Chinese policymakers should summarize and promote best practices from pilot areas, and gradually establish a nationwide strategic layout and integrated, collaborative innovation system for healthcare data. This would further facilitate the development of a robust national health information “artery.” Second, the results of heterogeneity analysis indicate that health improvement effects of the NHDC construction vary across different demographic groups. Governments should prioritize support for digital infrastructure in underdeveloped regions and extend digital care to vulnerable populations to bridge the digital divide. This would advance the inclusive development of high-quality, accessible healthcare data, enhancing the public’s sense of fulfillment, happiness, and security. Third, the mechanism analysis results underscore the importance of health management in improving health of older adults in the context of emerging digital health trends. In the future, healthcare data resources should be leveraged as key assets, and new health information technologies should be used as strong support to accelerate the interoperability of digital health into health management. This would help create a digital health management model tailored to the health needs of older adults.

### Limitations

This study has several limitations due to data constraints and space limitations. First, the study demonstrated statistical significance but failed to establish clinical relevance. The magnitude of effect sizes lacked contextualization against established clinical benchmarks. Second, the study failed to provide evidence demonstrating whether the NHDC’s infrastructure investment yields proportionate reductions in healthcare expenditures or quantifiable socioeconomic returns. Finally, although briefly discussed in the heterogeneity analysis, this study did not deeply explore whether the NHDC construction could exacerbate the “digital divide” and thereby affected health equity. These issues warrant further investigation.

## Conclusion

In this paper, we utilized data from the four waves of the CHARLS and employed the NHDC pilot project as a policy shock to construct a quasi-natural experiment. Using a difference-in-differences approach, we investigated the impact of NHDC construction on the health of older adults in pilot provinces, and we elaborated on the role of health management within the underlying mechanism.

The study finds that the NHDC construction significantly improves health outcomes of older adults. This improvement is reflected in higher subjective health ratings, reduced prevalence of chronic diseases and multimorbidity, enhanced physical function, and improved mental health. Further analysis indicates that this positive health effect is more significant among rural residents, individuals under 60 years of age, and male seniors. Mechanism analysis reveals that increased active health management efforts by the older adults, along with a higher likelihood of receiving health management services, play a significant role in the channels of effect.

## Data Availability

Publicly available datasets were analyzed in this study. This data can be found here: https://charls.pku.edu.cn.
